# Detection of Avian Influenza A(H7N9) Virus from Live Poultry Markets in Guangzhou, China: A Surveillance Report

**DOI:** 10.1371/journal.pone.0107266

**Published:** 2014-09-12

**Authors:** Zongqiu Chen, Kuibiao Li, Lei Luo, Enjie Lu, Jun Yuan, Hui Liu, Jianyun Lu, Biao Di, Xincai Xiao, Zhicong Yang

**Affiliations:** Guangzhou Center for Disease Control and Prevention, Guangzhou, Guangdong, China; The University of Hong Kong, Hong Kong

## Abstract

**Purpose:**

A virologic surveillance program for A(H7N9) virus was conducted from April 15, 2013 to February 14, 2014 in Guangzhou, aiming to clarify the geographical distribution of A(H7N9) viruses among live poultry markets (LPMs) and poultry farms in Guangzhou. Virological and serological surveys of poultry workers were also conducted to evaluate the risk of poultry-to-human transmission of the A(H7N9) virus.

**Methods:**

36 retail LPMs, 6 wholesale LPMs and 8 poultry farms were involved in our surveillance program. About 20 live poultry and environmental samples were obtained from each surveillance site at every sampling time. Different environmental samples were collected to represent different poultry-related work activities. RT-PCR and virus culture were performed to identify the A(H7N9) virus. Hemagglutinin inhibition assay and RT-PCR were conducted to detect possible A(H7N9) infection among poultry workers.

**Results:**

A total of 8900 live poultry and environmental samples were collected, of which 131(1.5%) were tested positive for A(H7N9) virus. 44.4% (16/36) of retail LPMs and 50.0% (3/6) of wholesale LPMs were confirmed to be contaminated. No positive samples was detected from poultry farms. A significant higher positive sample rate was found in environmental samples related to poultry selling (2.6%) and slaughtering (2.4%), compared to poultry holding (0.9%). Correspondingly, A(H7N9) viruses were isolated most frequently from slaughter zone. In addition, 316 poultry workers associated with the 19 contaminated-LPMs were recruited and a low seroprevalence (1.6%) of antibody against A(H7N9) virus was detected. An asymptomatic A(H7N9) infection was also identified by RT-PCR.

**Conclusions:**

Our study highlights the importance of conducting effective surveillance for A(H7N9) virus and provides evidence to support the assumption that slaughtering is the key process for the propagation of A(H7N9) virus in retail LPMs. Moreover, the ability of A(H7N9) virus to cross species barrier is proved to be still limited.

## Background

A novel reassortant avian influenza A(H7N9) virus, characterized by rapidly progressive pneumonia, development of acute respiratory distress syndrome and fatal outcomes [1], has emerged in China since February 2013 [2]. As of May 31, 2013, the first-wave outbreak resulted in 133 human cases [3], most of which were sporadically identified in Yangtze River Delta of east China [4]. After a period of quiescence from June to September 2013, during which only 2 laboratory-confirmed cases were reported, a second-wave outbreak of A(H7N9) has re-emerged in Pearl River Delta of south China since November 2013. As of April 7, 2014, a total of 420 laboratory-confirmed A(H7N9) cases have been reported in China, of which 102 cases were identified in Guangdong province.

South China has been considered to be an important origin or even the epicenter of influenza [5], as several avian influenza virus (AIV) subtypes crossing species barrier to infect human were firstly reported in this region [6], [7]. Guangzhou, the provincial capital of Guangdong province in south China, imports about 200,000 live poultry from neighboring cities daily. There are more than 600 live poultry markets (LPMs) in Guangzhou and about 80% of households in Guangzhou possess a habit of purchasing live poultry at LPMs [8]. The large amount of live poultry processed in LPMs daily, the high population density, and the cultural preference of purchasing freshly slaughtered poultry among households there make it an ideal environment for AIVs to be introduced, to transmit among different avian species, and even to infect humans. Over the past decade, AIV human infections have been occasionally reported in Guangzhou [9]–[13].

Food markets that offer both live poultry and fresh poultry meat either for sale or for slaughter are generally referred to as live poultry markets. Direct epidemiological link between LPMs and human infection has been established for avian influenza A(H7N9) virus. Poultry exposure, especially LPM exposure, is considered as the key risk factor for zoonotic transmission of A(H7N9) virus [14]–[17]. LPMs provide an optimal environment for zoonotic transfer and evolution of AIVs as they serve as the major contact points between humans and live poultry. Therefore, the maintenance of sensitive A(H7N9) virus surveillance in LPMs is of great importance since it can not only ensure early discovery and assessment of contamination level of the virus, but also make early detection of the substantial genetic mutation or virus reassortment possible, which will provide timely information for implementing proper control measures.

In response to the emerging A(H7N9) outbreak, a virologic surveillance program for A(H7N9) virus among various poultry premises has been established in Guangzhou since April, 2013. This report summarizes the results of the virologic surveillance program conducted from April 2013 to February 2014 and identifies the environmental sites commonly contaminated by A(H7N9) in LPMs. Virological and serological surveys conducted in poultry workers linked to A(H7N9)-contaminated LPMs and results of medical observations in them are also reported.

## Methods

### Selection of surveillance sites

**Figure 1:**
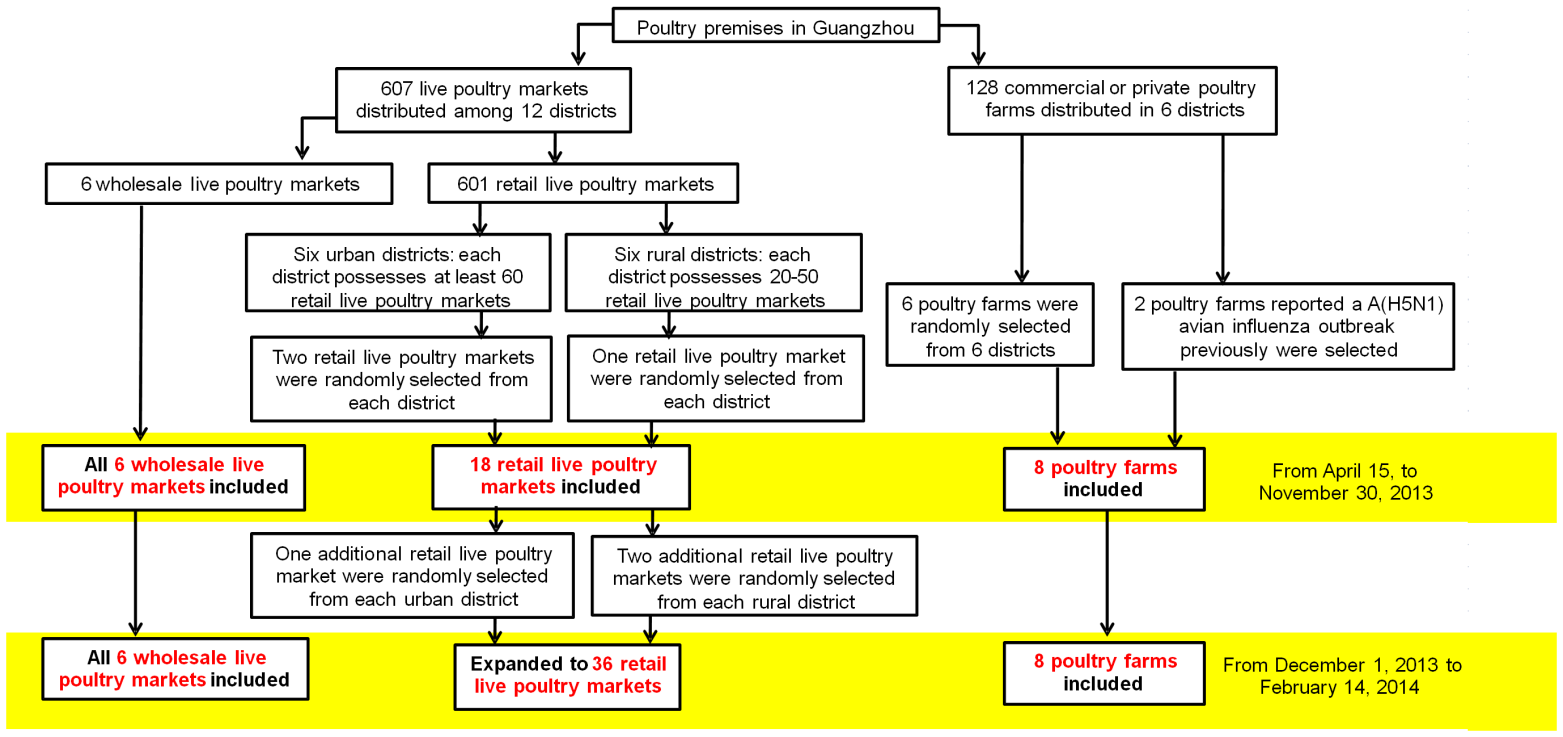
Enrollment of surveillance sites of the virologic surveillance program for A(H7N9) in Guangzhou, China.

As shown in Figure 1, 18 out of the total 601 retail LPMs in Guangzhou were randomly selected from all the 12 districts as surveillance sites using a random number table. Because a wholesale LPM always plays a key role in the poultry transaction chain, all the 6 wholesale LPMs in Guangzhou were involved. Among the 128 commercial or private poultry rearing farms in Guangzhou, 6 poultry farms were randomly selected from 6 districts as surveillance sites and 2 poultry farms with previous report of avian influenza A(H5N1) virus outbreak were also included. After two confirmed A(H7N9) cases were reported in Guangdong province [18], [19] and A(H7N9) viruses were detected from several LPMs in other cities of Guangdong (unpublished data), the number of retail LPMs selected as surveillance sites was subsequently expanded to 36 on December 1, 2013.

### Sample collection

About 4 live poultry stalls, selling different poultry species, were selected as sampling sites in each wholesale LPM at every sampling time. 5–6 samples, including tracheal and cloacal swabs of live poultry, as well as environmental samples of drinking water, fecal droppings and cage floors, were collected from each stall. Drinking water samples were collected from water trough, which were shared by all the poultry in the same cage, while fecal samples were collected from 3 to 5 fresh fecal droppings. Similarly, 10–20 samples were collected from each poultry farm at every sampling time.

As for a retail LPM, which commonly holds 4–6 live poultry stalls, 2 live poultry stalls were selected as sampling sites at every sampling time. About 10 environmental samples were collected from each stall. Different samples were collected to represent different poultry-related work activities: floor swabs in delivery area and cage swabs on truck were related to delivery of poultry into LPMs; fecal droppings swabs, poultry cage swabs and drinking water samples were related to poultry holding; visceral waste and waste bin swabs, scalding machine swabs, bloody sewage samples and swabs of floor in slaughter area were related to slaughter process; chopping boards swabs and swabs of tables for poultry display were related to sale process.

The virologic surveillance program for A(H7N9) virus was divided into three phases based on the difference in sampling frequency. Each surveillance site was sampled biweekly in the first phase from April 15 to May 30, 2013. With closure of LPMs and warmer weather, the first-wave outbreak of A(H7N9) virus was apparent halted in May, 2013 in China. The sampling frequency in the second phase from June 1 to November 30, 2013 was subsequently changed to every two months. From December 1, 2013, the sampling frequency in the third phase was changed back to biweekly because of the emerging outbreak of A(H7N9) in Guangdong province. The surveillance program was ceased on February 14, 2014 due to a mandatory requirement issued by the authority for temporary closure of all LPMs in Guangzhou.

### Information of surveillance sites

Through reviews of transport and sales records of LPMs, visual inspection of each surveillance site and interviews with managers and stallholders of each participating LPM and farmers of each participating poultry farm, information about volume of poultry in the LPM and poultry farm, the source of poultry, the infrastructure of poultry stalls, the general demarcation of work flow and activities related to poultry in the LPM and poultry farm, the health status of the poultry and the routine cleaning and disinfection procedure of the LPM and poultry farm was obtained by officers of local Center of Disease Control and Prevention using a standardized questionnaire.

### Medical observation and the virological and serological survey of poultry workers

At the time when each surveillance site was investigated and sampled, a written consent was obtained from all the associated poultry workers of the LPM or the poultry farm and a standardized questionnaire was used to collect information about their demographic characteristics, previous health status and immunization history, duration and frequency of poultry exposure, pattern of exposure, personal protective equipment and hygienic practices.

In this study, a A(H7N9)-contaminated LPM or poultry farm was defined as a LPM or a poultry farm that at least one environmental sample or poultry sample was tested positive for A(H7N9) virus. Once a surveillance site was identified to be contaminated by A(H7N9) virus, the associated poultry workers would be immediately recruited and throat swabs would also be collected from all of them within 24 hours for detection of probable human A(H7N9) infection, following by a seven-day medical observation to monitor the development of any symptoms. A second throat swab would be collected if a poultry worker developed symptoms of influenza-like-illness or pneumonia during seven-day medical observation. A serum sample would also be collected from all the associated poultry workers at the end of the seven-day medical observation.

### Laboratory tests

Poultry samples, environmental samples and throat swabs were collected in Universal transport medium (produced by Copan Italia), which included 3 ml combined viral transport medium in a 16×100 mm tube and a regular flocked swab. All samples were stored at 4°C and transported to laboratory of Guangzhou CDC within 24 hours. Viral RNA was extracted from the specimens using the RNeasy Mini Kit (QIAGEN, Germany). Real-time reverse-transcrip-tase-polymerase-chain-reaction (RT-PCR) using pairs of H7- and N9-specific primers provided by the Chinese National Influenza Center [20], was performed to detect A(H7N9) viral RNA. Samples were considered positive if the cycle threshold was ≤38.0 following protocol recommendations. Samples tested positive for A(H7N9) viral RNA were inoculated to the allantoic sac of 10-day-old pathogen-free embryonated chicken eggs and incubated for 48 to 72 hours at 35°C for viral culture.

Serum samples were prepared using standard procedures and then treated with receptor-destroying enzyme (RDE) from Vibrio cholerae (Denka Seiken) for 18 h at 37°C and inactivated for 30 minutes at 56°C. Hemagglutinin inhibition (HI) assay using horse red blood cells was conducted to detect antibodies against influenza A(H7N9) virus following laboratory procedures issued by World Health Organization [21]. Antigens for the HI assay were produced from the A/Guangzhou/1/2014(H7N9) strain (GenBank accession nos.KJ415822) isolated from a laboratory-confirmed A(H7N9) case in Guangzhou.

### Statistical method

Data were entered into a customized database (Microsoft Excel 2007) and then transferred into a SPSS (Version 13.0) for analysis. Description statistics were used to calculate distributions of all variables. Chi-squared tests (α = 0.05) and Fisher exact tests were used to compare the positive sample rate among different phases of surveillance, different poultry premises, different sample types and different poultry-related work activities.

### Ethics statement

Our study was reviewed and approved by the Ethics Committee of Guangzhou Center for Disease Control and Prevention. All poultry workers in this study had provided their written consent to participate when investigators began the interviews. All human data were anonymized. Sampling activity of poultry in this study was approved by the Ethics Committee of Guangzhou Center for Disease Control and Prevention and the Commerce Bureau of Guangzhou City. Permissions from owners of poultry farms to collect samples from their animals were also obtained. No endangered or protected species were involved in our study.

## Results

### LPM practices and poultry source

All LPMs involved in this virologic surveillance program operated daily to sell chickens, ducks, geese and pigeons. In contrast, quails and wild birds were only observed in 3 and 2 LPMs, respectively. Chicken was the most common species in LPMs and accounted for about 60% of all poultry. We observed that chickens, ducks, geese and pigeons were always mixed together in the same poultry stall for sale and even in the same cage among some stalls in retail LPMs. In contrast, a poultry stall of a wholesale LPM or a poultry farm commonly possessed only one kind of poultry. 31 (86.0%) retail LPMs and all wholesale LPMs reported keeping live poultry overnight for 2–4 days until sold. Ducks, geese and pigeons stayed 3 times longer than chickens on average. Slaughtering live poultry was observed in all 36 retail LPMs while the 6 wholesale LPMs were for the trading of whole poultry without slaughtering process. All the 42 LPMs reported washing poultry zones daily and 23 (55%) reported applying detergent or disinfectant daily with respect to cleaning and disinfection.

**Figure 2:**
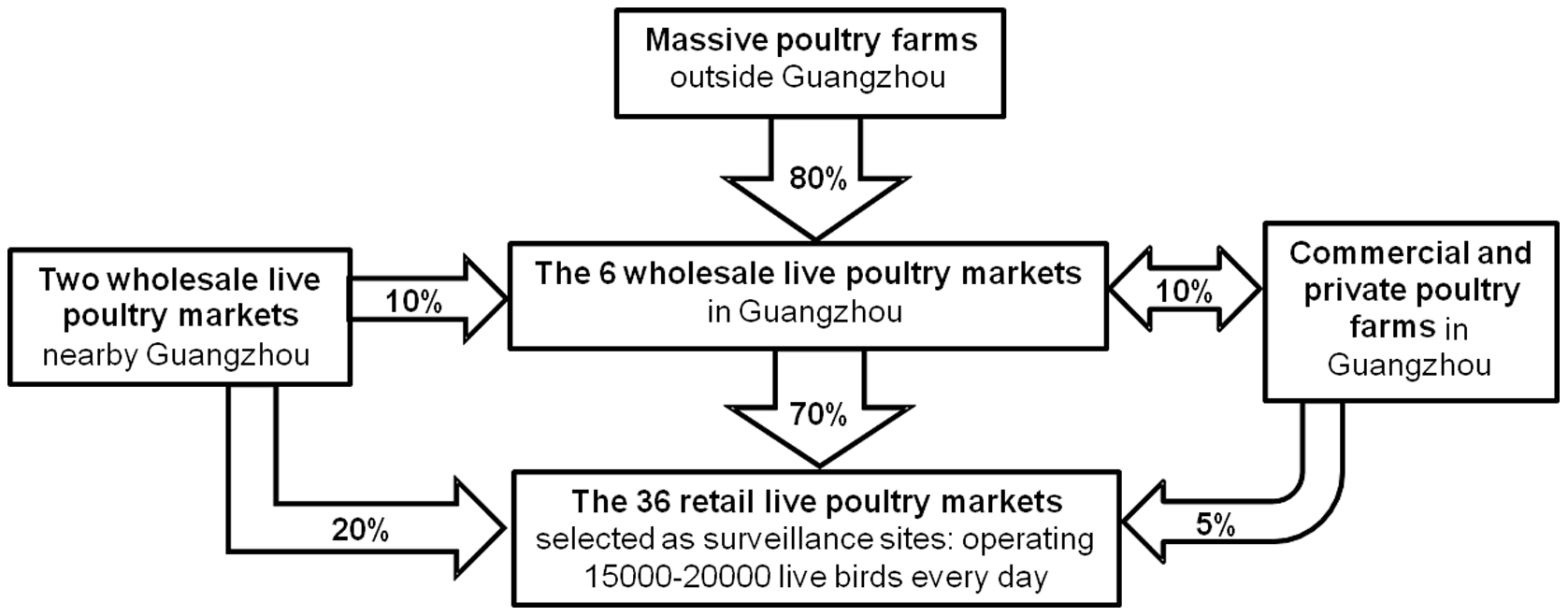
The general routes of live poultry transmission in Guangzhou, China.

According to our investigation and a review of transport and sales records, 400–600 live birds were slaughtered and sold daily (80–100 for each live poultry stall) in a retail LPM. Therefore, a total of 15000–20000 live poultry were processed in the 36 retail LPMs selected as surveillance sites every day. Tracing back from the poultry source of those retail LPMs, about 70% of the poultry were supplied by the 6 wholesale LPMs in Guangzhou, 20% were received from the other two wholesale LPMs nearby Guangzhou (Figure 2), and the remaining 10% were directly imported from poultry rearing farms in Guangzhou (5%) or nearby Guangzhou(5%). Furthermore, the poultry source of the 6 wholesale LPMs in Guangzhou could be traced back to a large number of poultry rearing farms outside Guangzhou (about 80%), a few poultry rearing farms in Guangzhou (about 10%) and the other two wholesale LPMs nearby Guangzhou (10%).

### Laboratory findings of virologic surveillance

**Figure 3:**
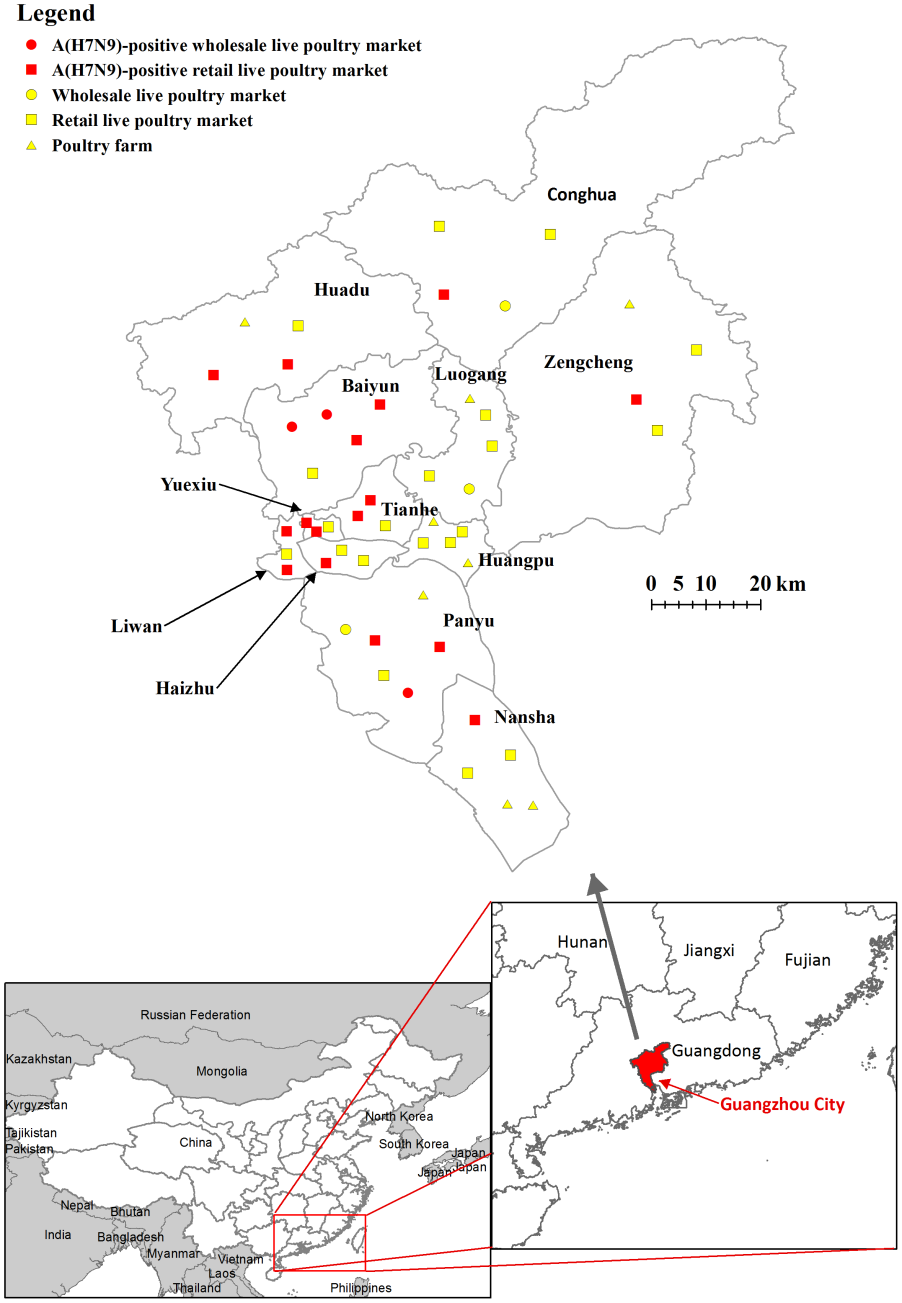
Geographic distribution of surveillance sites of the virologic surveillance program for A(H7N9) in Guangzhou, China, April 15, 2013 to February 14, 2014.

From April 1, 2013 to February 14, 2014, a total of 8900 live poultry and environmental samples were collected from the 50 surveillance sites in Guangzhou. Overall, 131(1.5%) samples collected from 16 retail LPMs and 3 wholesale LPMs were tested positive for A(H7N9) virus by RT-PCR (Figure 3). 44.4% (16/36) of retail LPMs and 50.0% (3/6) of wholesale LPMs were confirmed to be contaminated (Table 1). No sample from poultry farms was tested positive. 97.9% (128/131) of the A(H7N9)-positive samples were collected from December 28, 2013 to February 14, 2014 (Table 1).

**Table 1:** Monthly results of virologic surveillance for A(H7N9) virus in retail live poultry markets, wholesale live poultry markets and poultry farms in Guangzhou, China, April 2013–February 2014.

Month	Retail LPMs		Wholesale LPMs		Poultry farms		Total	
	Positive rate of samples (Positive samples/Sample size)	Positive rate of LPMs (Positive LPMs/LPMs sampled)	Positive rate of samples (Positive samples/Sample size)	Positive rate of LPMs (Positive LPMs/LPMs sampled)	Positive rate of samples (Positive samples/Sample size)	Positive rate of Poultry farms (Positive farms/Farms sampled)	Positive rate of samples (Positive samples/Sample size)	Positive rate of Surveillance sites (Positive sites/Total sites)
**2013-Apr**	0.0% (0/601)	0.0% (0/18)	0.0% (0/140)	0.0% (0/6)	0.0% (0/225)	0.0% (0/8)	0.0% (0/966)	0.0% (0/32)
**2013-May**	**0.3% (3/902)**	**5.6% (1/18)**	0.0% (0/340)	0.0% (0/6)	0.0% (0/415)	0.0% (0/8)	**0.2% (3/1657)**	**3.1% (1/32)**
**2013-Jun**	0.0% (0/181)	0.0% (0/9)	0.0% (0/84)	0.0% (0/4)	0.0% (0/60)	0.0% (0/4)	0.0% (0/325)	0.0% (0/17)
**2013-Jul**	0.0% (0/160)	0.0% (0/8)	0.0% (0/60)	0.0% (0/3)	0.0% (0/60)	0.0% (0/4)	0.0% (0/280)	0.0% (0/15)
**2013-Aug**	0.0% (0/180)	0.0% (0/9)	0.0% (0/60)	0.0% (0/3)	0.0% (0/60)	0.0% (0/4)	0.0% (0/300)	0.0% (0/16)
**2013-Sep**	0.0% (0/181)	0.0% (0/9)	0.0% (0/62)	0.0% (0/3)	0.0% (0/60)	0.0% (0/4)	0.0% (0/303)	0.0% (0/16)
**2013-Oct**	0.0% (0/180)	0.0% (0/9)	0.0% (0/60)	0.0% (0/3)	0.0% (0/60)	0.0% (0/4)	0.0% (0/300)	0.0% (0/16)
**2013-Nov**	0.0% (0/200)	0.0% (0/10)	0.0% (0/106)	0.0% (0/4)	0.0% (0/60)	0.0% (0/4)	0.0% (0/366)	0.0% (0/18)
**2013-Dec**	**0.4% (3/678)**	**2.8% (1/36)**	0.0% (0/180)	0.0% (0/6)	0.0% (0/160)	0.0% (0/8)	**0.3% (3/1018)**	**2.0% (1/50)**
**2014-Jan**	**5.6% (89/1584)**	**41.7% (15/36)**	**1.3% (4/300)**	**16.6% (1/6)**	0.0% (0/228)	0.0% (0/8)	**4.4% (93/2112)**	**32.0% (16/50)**
**2014-Feb**	**2.8% (24/845)**	**33.3% (12/36)**	**3.3% (8/240)**	**33.3% (2/6)**	0.0% (0/188)	0.0% (0/8)	**2.5% (32/1273)**	**28.0% (14/50)**
**Total**	**2.1% (119/5692)**	**44.4% (16/36)**	**0.7% (12/1632)**	**50.0% (3/6)**	**0.0% (0/1576)**	**0.0% (0/8)**	**1.5% (131/8900)**	**38.0% (19/50)**

The first surveillance site confirmed to be contaminated by A(H7N9) virus was a retail LPM in a rural district. A chopping boards swab out of the 20 samples obtained from that LPM on May 16, 2013 was tested positive for A(H7N9) virus on the next day. A strengthening sampling was implemented on May 17 to investigate the extent of A(H7N9)-contamination and 63 additional samples were collected from the market. Subsequently, A cloacal swab of chicken and a bloody sewage sample were also tested positive for A(H7N9) virus on May 18. A wholesale LPM (involved in our surveillance) providing live poultry to the A(H7N9)-contaminated retail LPM was traced. However, neither the 60 samples collected from the wholesale LPM previously nor the 292 samples collected on May 18 showed any positive result of A(H7N9) virus.

After the detection of A(H7N9) virus in a retail LPM on May 16, 2013, no further detection of A (H7N9) virus in LPMs or poultry farms was recorded for over six months in Guangzhou (Table 1), until another retail LPM was confirmed to be contaminated on December 31, 2013. From then on, repeated detection of A (H7N9) virus in LPMs was reported in Guangzhou. The result of chi-squared test indicated that the positive sample rate in the third phase of surveillance (2.9%) was significantly higher (P<0.001) than that in the first (0.1%) and second (0.0%) phase (Table 2).

**Table 2:** Results of the three periods of virologic surveillance for A(H7N9) virus in live poultry markets and poultry farms in Guangzhou, China, April 2013–February 2014.

Characteristic	The first phase of surveillance (April 15 to May 31, 2013)		The second phase of surveillance (June 1 to November 31, 2013)		The third phase of surveillance (December 1, 2013 to February 14, 2014)		Total		P-value of Chi-squared test
	Sample size	Positive samples (%)	Sample size	Positive samples (%)	Sample size	Positive samples (%)	Sample size	Positive samples (%)	
**Poultry premises category**									**P<0.001**
Retail LPMs	1503	3(0.2%)	1082	0(0%)	3107	116(3.7%)	5692	119(2.1%)	
Wholesale LPMs	480	0(0%)	432	0(0%)	720	12(1.7%)	1632	12(0.7%)	
Poultry rearing farms	640	0(0%)	360	0(0%)	576	0(0%)	1576	0(0%)	
**Sample types**									**P<0.001**
** Tracheal and cloacal swabs**	**1124**	**1(0.1%)**	**402**	**0(0%)**	**516**	**8(1.6%)**	**2042**	**9(0.4%)**	
Chicken	540	1(0.2%)	220	0(0%)	328	7(2.1%)	1088	8(0.7%)	
Duck	197	0(0%)	66	0(0%)	72	0(0%)	335	0(0%)	
Goose	119	0(0%)	48	0(0%)	40	1(2.5%)	207	1(0.5%)	
Pigeon	140	0(0%)	28	0(0%)	20	0(0%)	188	0(0%)	
Quail	46	0(0%)	16	0(0%)	20	0(0%)	82	0(0%)	
Wild birds	82	0(0%)	24	0(0%)	36	0(0%)	142	0(0%)	
** Environmental samples of different poultry-related work activities**	**1499**	**2(0.1%)**	**1472**	**0(0%)**	**3887**	**120(3.1%)**	**6858**	**122(1.8%)**	**P<0.001**
** Poultry delivering**	**0**	**0(0%)**	**0**	**0(0%)**	**201**	**7(3.5%)**	**201**	**7(3.5%)**	**P<0.001**
Floor swab in delivery area	0	0(0%)	0	0(0%)	46	0(0%)	46	0(0%)	
Cage swab on truck	0	0(0%)	0	0(0%)	155	7(4.5%)	155	7(4.5%)	
** Poultry holding**	**888**	**0(0%)**	**686**	**0(0%)**	**1726**	**31(1.8%)**	**3300**	**31(0.9%)**	**P<0.001**
Fecal droppings swab	459	0(0%)	349	0(0%)	824	14(1.7%)	1632	14(0.9%)	
Poultry cage swab	331	0(0%)	224	0(0%)	506	7(1.4%)	1061	7(0.7%)	
Drinking water sample	98	0(0%)	113	0(0%)	396	10(2.5%)	607	10(1.6%)	
** Poultry slaughtering**	**341**	**1(0.3%)**	**477**	**0(0%)**	**1009**	**43(4.3%)**	**1827**	**44(2.4%)**	**P<0.001**
Scalding machine swab	114	0(0%)	98	0(0%)	224	7(3.1%)	436	7(1.6%)	
Visceral waste and waste bin swab	117	0(0%)	127	0(0%)	286	12(4.2%)	530	12(2.3%)	
Bloody sewage sample	110	1(0.9%)	166	0(0%)	347	18(5.2%)	623	19(3%)	
Swab of floor in slaughter area	0	0(0%)	86	0(0%)	152	6(3.9%)	238	6(2.5%)	
** Poultry selling**	**270**	**1(0.4%)**	**309**	**0(0%)**	**951**	**39(4.1%)**	**1530**	**40(2.6%)**	**P = 0.394**
Chopping boards swab	178	1(0.6%)	199	0(0%)	636	27(4.2%)	1013	29(2.9%)	
Swab of tables for poultry display	92	0(0%)	110	0(0%)	315	12(3.8%)	517	11(2.1%)	
**Total**	**2623**	**3(0.1%)**	**1874**	**0(0%)**	**4403**	**128(2.9%)**	**8900**	**131(1.5%)**	* **P<0.001**

*Chi-squared test of positive sample rate among the three phase of surveillance.

As showed in Table 2, the positive sample rate was highest (P<0.001) in retail LPMs (2.1%) among different poultry premises, followed by wholesale LPMs (0.7%) and poultry farms (0.0%). A significant higher (P<0.001) positive sample rate was found in environmental samples (1.8%) than live poultry samples (0.4%), and in environmental samples related to poultry selling (2.6%) and slaughtering (2.4%), compared to poultry holding (0.9%). Among samples related to poultry slaughtering and selling, bloody sewage samples (3.0%) and chopping boards swabs (2.9%) were found most heavily contaminated in the slaughter zone and sale zone respectively. Among samples related to poultry holding, the positive sample rate in fecal droppings swabs (0.9%) was significantly lower (P<0.001) than that in drinking water samples (1.6%). Another commonly contaminated site was poultry cage on truck in delivery zone, with a positive rate of 4.5% (Table 2).

Of the 131 samples tested positive for A(H7N9) viral RNA, 23 A(H7N9) viruses (17.6%) were successfully isolated. All isolated viruses came from 9 retail LPMs and 2 wholesale LPMs, where 1–3 viruses were isolated per LPM. A(H7N9) viruses were most frequently isolated from environmental samples collected from slaughter zone (11/23), especially those bloody sewage samples (6/11). In addition, A(H7N9) viruses were also isolated from visceral waste and waste bin swabs (4/23), chopping boards swabs (4/23), drinking water samples (3/23), tracheal and cloacal swabs of chickens (3/23), fecal droppings swabs (2/23) and swabs of floor in slaughter area(1/23).

### Virological and serological surveys of poultry workers

A total of 316 poultry workers associated with the 19 A(H7N9)-contaminated LPMs were recruited. Their median age was 40.8 years (rang 18–65 years) and 56.6% of them were male. 84.5% poultry workers were from 16 retail LPMs and the rest were from 3 wholesale LPMs. All of them reported a history of direct poultry exposure (average 9.6 years) while only 9.0% reported using masks and gloves when they were working in LPMs. A few workers (2.1%) reported history of influenza A vaccination.

The poultry workers were monitored for seven days, during which 321 throat swabs were collected (six workers who developed symptoms during medical observation were collected twice). Only one throat swab was positive for A(H7N9) virus by RT-PCR and an asymptomatic human A(H7N9) infection was thus detected. The infected poultry worker was a 59 year-old male without any chronic disease. He managed a live poultry stall in a retail LPM, having direct contact with live poultry every day without any personal protective equipment. Besides occupational exposure, neither other route of poultry exposure nor contact with sick people was reported. Two environmental samples of his stall were tested positive for A(H7N9) virus on January 27, 2014. His throat swab collected on January 28 was also positive for A(H7N9), although no symptoms was developed. As the three throat swabs collected on 30, 31 January and 6 February were consecutively tested negative, and no symptoms or abnormalities were revealed from his chest radiography, the worker was subsequently discharged.

Of the 316 serum samples collected from these poultry workers, 1.6% (5/316) reported influenza A(H7N9) antibody titers over 40 (2 had an HI titer of 80) as detected by HI assay, including the serum sample collected from the asymptomatic infected poultry worker detected by RT-PCR (HI titer of 80). The median age of the 5 serologic-positive poultry workers was 46.6 years (rang 40–59 years) and three of them were male. All workers were working on a retail LPM and reported a history of direct contact with poultry every day, especiallyslaughtering and de-feathering poultry. None of them reported any development of respiratory symptoms during the past month.

## Discussion

The fact that pathogenicity of A(H7N9) virus is significant to human but low to poultry [22] highlight the importance of effective and systematic surveillance in various poultry premises. From analysis of the 11-months virologic surveillance program for A(H7N9) virus in Guangzhou, we clarify the long term trend of the geographical distribution of avian influenza A(H7N9) viruses among LPMs. We alsoreveal that the routes of A(H7N9) virus transmission in Guangzhou are mainly from poultry farms outside Guangzhou to the 6 wholesale LPMs in Guangzhou, and then to massive retail LPMs in Guangzhou. Thus our results suggest that conducting virologic surveillance for A(H7N9) virus in various poultry premises is an efficient way to provide insight into the mechanisms of spread of A(H7N9) and baseline information for informing targeted control measures. Furthermore, a significant higher positive rate of A(H7N9) viral RNA was observed in environmental samples rather than live poultry samples by chi-squared tests, suggesting that collecting environmental samples rather than live poultry samples is an more efficient way to conduct A(H7N9) virus surveillance.

Extensive environmental contamination of A(H7N9) virus were detected in LPMs in Guangzhou through our surveillance and 23 A(H7N9) viruses were successfully isolated. More importantly, only 3 samples from a retail LPM out of the total 4497 samples collected from 42 LPMs during April 15 to November 31, 2013 were tested positive for A(H7N9) virus, indicating a very low degree of A(H7N9) contamination of LPMs in Guangzhou that time. This finding echoes the fact that there was no confirmed A(H7N9) human case reported in Guangzhou at that time. It was only after A(H7N9) viruses began to be frequently detected from various environmental sites of LPMs since December 31, 2013 that the first A(H7N9) human case of Guangzhou was identified on 10 January 2014 [23], followed by another 20 cases confirmed as of March 7, 2014. We thus provide evidence to support the idea that LPMs are reservoirs of A(H7N9) virus and locations of human A(H7N9) infection sources, based on the history of laboratory-confirmed A(H7N9) cases of poultry exposure [4], [15] and the high similarities between viral RNA sequences detected in human and LPMs [17], [24]–[26].

In our study, by chi-squared tests, a significant higher positive rate of A(H7N9) viral RNA was found in samples collected from retail LPMs than wholesale LPMs. As retail other than wholesale LPMs are the place where live poultry are slaughtered every day, the results imply that slaughtering maybe the key process to expand the extent of contamination from poultry to the entire environment in a live poultry stall. This assumption is further supported by our surveillance finding that environmental contamination with A(H7N9) virus was common in retail LPMs and the most commonly contaminated environmental sites were located in slaughter zone and sales zone. In addition, a significant higher positive rate of A(H7N9) viral RNA was detected in bloody sewage samples and chopping boards swabs by chi-squared tests, and A(H7N9) viruses were isolated most frequently from environmental samples collected from slaughter zone. This finding is not surprising as droplets that may contain viral particles are generated during the process of poultry slaughtering (including catching, slaughtering, defeathering and eviscerating). The secretions, internal tissues and organs with potential high viral loads can also widely splash out and diffuse to the narrow and poorly ventilated space of a poultry stall. If sufficient contaminated aerosolised material is inhaled by people who visit a contaminated LPM, an airborne transmission of A(H7N9) virus can occur. Our findings suggest that the practice of slaughtering live poultry (always in view of the customers) in LPMs should be discouraged to reduce the infection risk of LPM visitors and centralized poultry slaughtering factory should be considered as an alternative.

Our serologic survey in the poultry workers from A(H7N9)-contaminated LPMs indicated a low seroprevalence (1.6%) of antibody against A(H7N9) virus. Similar subclinical A(H7N9) infections of poultry workers based on serologic test of a single serum sample was reported previously [27]. However, the possibility of cross-reactivity with other antigenically similar influenza A(H7) viruses can not be ruled out. Given the fact that one case of human infection of A(H7N9) virus in a serologic-positive asymptomatic poultry worker was identified by RT-PCR on throat swabs in our study, we provide strong evidence that subclinical A(H7N9) infection did occur. Nevertheless, the fact that 98.4% of poultry workers involved in our survey remained infection-free suggests that the infection risk in poultry workers is low. Further studies are needed to investigate the potential role of pre-existing cross-reactive immunity that might be induced by exposure to other circulating AIVs already exist in LPMs in poultry workers. Take into considerations the low seroprevalence of antibody in poultry workers while LPMs in Guangzhou have been extensively contaminated by A(H7N9) virus since January, 2014 and thousands of people visit these LPMs every day, the relative low number of A(H7N9) cases indicates that the ability of virus to cross species barrier is still limited.

Our study has some limitations. First, in the source tracing investigation of the first A(H7N9)-contaminated LPM identified in Guangzhou on May 16, 2013, we missed the best time to collect samples from the wholesale LPM supplying poultry for the A(H7N9)-contaminated stall because disinfection had been performed before our sampling and those associated chickens had sold out. The wholesale LPM thus could not be clearly confirmed as the infection source due to lack of virologic evidence. However, according to the transport and sales records of the wholesale LPM, we believe that those infected chickens were introduced from a commercial poultry farm in a region where A(H7N9)-contaminated LPMs and A(H7N9) human cases were identified at that time. Second, take into considerations the large number of LPMs and large amount of poultry in Guangzhou, the number of retail LPMs selected as surveillance sites was relatively small before it expanded to 36 on December 1, 2013. Therefore, we cannot completely exclude the possibility that more LPMs could be contaminated by A(H7N9) from April to November 2013, thus the degree and extent of A(H7N9) virus contamination in Guangzhou can be undervalued. However, given the fact that no human A(H7N9) case was identified through intensive test of massive suspected pneumonia patients in Guangzhou in 2013 and the surveillance in other cities of Guangdong province also reported a similar low positive contamination rate in LPMs (unpublished data), it is unlikely that the virus was widely spread in Guangzhou at that time. Last, we did not conduct a more sensitive microneutralization test to detect antibody to A(H7N9) virus in poultry workers, which should be considered in the future.

## Conclusions

In conclusion, our study highlights the importance of conducting effective virologic surveillance for A(H7N9) virus in various poultry premises and suggests that the extensively contaminated LPMs are associated with the recent emerging human infections with A(H7N9) viruses in Guangzhou. The common contaminated environmental sites in LPMs identified in this study can establish the basis of future environmental sampling strategy for A(H7N9) surveillance in LPMs. We also provide evidence to support the idea that slaughtering is the key process for the propagation of A(H7N9) virus in retail LPMs, thus help to take targeted control measure to reduce infection risk of A(H7N9) in retail LPMs. Future work is required to further evaluate the effects of interventions in LPMs, such as the temporarily closure of all LPMs in Guangzhou since February 15, 2014.
